# Editorial: Monoclonal antibodies in treating multiple sclerosis (MS), and related diseases

**DOI:** 10.3389/fneur.2026.1925664

**Published:** 2026-09-30

**Authors:** Abdorreza Naser Moghadasi, Sreeram Ramagopalan, Simona Bonavita, Mahsa Ghajarzadeh

**Affiliations:** 1 Multiple Sclerosis Research Center, Neuroscience Institute, Tehran University of Medical Sciences, Tehran, Iran; 2 Centre for Pharmaceutical Medicine Research, King's College London, London, United Kingdom; 3 Dipartimento di Scienze Mediche e Chirurgiche Avanzate, Università della Campania Luigi Vanvitelli, Naples, Italy; 4 Department of Neurology, Johns Hopkins University School of Medicine, Baltimore, MD, United States

**Keywords:** anti-CD20, B-cell depletion, MOGAD, monoclonal antibody, multiple sclerosis, natalizumab, neuromyelitis optica spectrum disorder, ocrelizumab

Multiple sclerosis (MS) is a chronic immune-mediated neuroinflammatory disorder of the central nervous system (CNS) that disproportionately affects women of reproductive age and whose prevalence continues to rise globally. Although MS was traditionally regarded as a predominantly T-cell–mediated disease, growing evidence has established a central role for B cells in disease pathogenesis. This evolving understanding has driven the development of monoclonal antibody therapies targeting CD20-positive B cells, fundamentally transforming the management of MS and related CNS demyelinating disorders, including neuromyelitis optica spectrum disorder (NMOSD) and myelin oligodendrocyte glycoprotein antibody-associated disease (MOGAD).

This Research Topic brings together 12 contributions that collectively examine the mechanisms, efficacy, safety, and real-world application of monoclonal antibodies across the spectrum of CNS demyelinating diseases. The Research Topic encompasses mechanistic reviews, clinical trial analyses, retrospective cohort studies, imaging biomarker investigations, and illustrative case reports, providing a comprehensive overview of current advances in the field.

Several contributions deepen our understanding of disease biology and therapeutic mechanisms. Zhang and Li reviewed insights derived from experimental autoimmune encephalomyelitis (EAE) models and their implications for MOGAD pathogenesis. Their work highlights the unique pathogenic role of MOG-IgG antibodies and underscores the therapeutic potential of emerging strategies such as complement inhibition and plasma-cell depletion.

The evolving landscape of anti-CD20 therapies is explored through both clinical and real-world perspectives. Berkovich et al. reported a case series demonstrating that switching between anti-CD20 agents may remain an effective strategy for patients with inadequate response or tolerability concerns, while Fernandes et al. showed that long-term anti-CD20 treatment is associated with lymphopenia and hypogammaglobulinemia, emphasizing the need for ongoing immunologic surveillance despite a low incidence of serious adverse events.

The search for biomarkers capable of guiding individualized treatment is addressed by Rodríguez-Jorge et al., who demonstrated normalization of serum neurofilament light chain and glial fibrillary acidic protein among patients achieving no evidence of disease activity (NEDA) under ocrelizumab therapy. These findings support the utility of fluid biomarkers in distinguishing inflammatory disease control from progression driven by neurodegenerative mechanisms.

Evidence supporting the efficacy of newer monoclonal antibodies was further strengthened by Alvarez et al., whose pooled analyses of the phase 3 ULTIMATE I and II trials demonstrated consistently higher rates of NEDA with ublituximab compared with teriflunomide across a broad range of patient subgroups. Notably, new or enlarging T2 lesions during the first 6 months of treatment emerged as the principal contributor to residual disease activity, providing clinically relevant insights into the expected timeline of therapeutic response.

Two studies focused on natalizumab provided complementary perspectives on treatment outcomes. Puthenparampil, Scialpi et al. demonstrated greater improvements in cognitive processing speed among pediatric-onset MS patients compared with adult-onset patients, supporting the use of natalizumab in younger populations at risk for early cognitive impairment. In a separate investigation, Puthenparampil, Basili et al. used optical coherence tomography to assess retinal hyper-reflective foci, revealing persistent compartment-specific microglial activity despite effective blockade of peripheral leukocyte trafficking.

The Research Topic also highlights important advances in NMOSD. Zhou et al. reported superior disease control and treatment persistence with rituximab compared with conventional oral immunosuppressants in a multicenter Chinese cohort, while Li et al. described successful use of the anti-CD19 monoclonal antibody inebilizumab in a patient with refractory NMOSD complicated by Sjögren's syndrome and pancytopenia, suggesting broader therapeutic implications for B-cell–driven autoimmune disorders.

Beyond relapse prevention, Wang et al. explored the neural correlates of neuropathic pain in NMOSD using resting-state functional MRI, identifying alterations in amygdalar and insular activity associated with pain severity and duration. These findings provide valuable insights into symptom mechanisms that extend beyond inflammatory lesion formation and remain inadequately addressed by current immunotherapies.

Finally, Xue et al. presented the first reported pediatric Chinese case of Guillain–Barré syndrome with bilateral optic neuritis successfully treated with rituximab after failure of conventional therapies. This report illustrates both the diagnostic challenges posed by overlapping central and peripheral demyelinating syndromes and the expanding therapeutic role of B-cell depletion in rare neuroimmunological disorders.

Collectively, the studies included in this Research Topic demonstrate that monoclonal antibodies have evolved beyond therapeutic agents to become powerful tools for elucidating disease mechanisms, identifying biomarkers, and refining personalized treatment strategies. Several overarching themes emerge, including the value of within-class switching among anti-CD20 therapies, the importance of long-term safety monitoring, the persistent efficacy gap between biologics and traditional immunosuppressants, and the need to address residual symptoms such as cognitive dysfunction and neuropathic pain. Together, these contributions advance our understanding of MS, NMOSD, and MOGAD and provide a foundation for future research aimed at optimizing the selection, sequencing, and long-term use of monoclonal antibody therapies in neuroimmunology.

